# Discrimination study between carcass yield and meat quality by gender in Korean native cattle (Hanwoo)

**DOI:** 10.5713/ajas.19.0472

**Published:** 2019-08-26

**Authors:** Do-Gyun Kim, Joon-Yong Shim, Byoung-Kwan Cho, Collins Wakholi, Youngwook Seo, Soohyun Cho, Wang-Hee Lee

**Affiliations:** 1 Department of Biosystems Machinery Engineering, Collage of Agricultural and Life Science, Chungnam National University, Daejeon 34134, Korea; 2 Agricultural Bigdata Division, Rural Development Administration, Jeonju 54875, Korea; 3 National Institute of Agricultural Sciences, Rural Development Administration, Jeonju 54875, Korea; 4 Animal Products Utilization Division, National Institute of Animal Science, Rural Development Administration, Wanju 55365, Korea

**Keywords:** Hanwoo, Carcass Grade, Carcass Yield, Meat Quality, Discriminant Function Analysis

## Abstract

**Objective:**

The aim of this study was to identify a distribution pattern of meat quality grade (MQG) as a function of carcass yield index (CYI) and the gender of Hanwoo (bull, cow, and steer) to determine the optimum point between both yield and quality. We also attempted to identify how pre- and post-deboning variables affect the gender-specific beef quality of Hanwoo.

**Methods:**

A total of 31 deboning variables, consisting of 7 pre-deboning and 24 post-deboning variables from bulls (n = 139), cows (n = 69), and steers (n = 153), were obtained from the National Institute of Animal Science (NIAS) in South Korea. The database was reconstructed to be suitable for a statistical significance test between the CYI and the MQG as well as classification of meat quality. Discriminant function analysis was used for classifying MQG using the deboning parameters of Hanwoo by gender.

**Results:**

The means of CYI according to 1+, 1, 2, and 3 of MQG were 68.64±2.02, 68.85± 1.94, 68.62±5.88, and 70.99±3.32, respectively. High carcass yield correlated with low-quality grade, while high-quality meat most frequently was obtained from steers. The classification ability of pre-deboning parameters was higher than that of post-deboning parameters. Moisture and the shear force were the common significant parameters in all discriminant functions having a classification accuracy of 80.6%, 71%, and 56.9% for the bull, cow, and steer, respectively.

**Conclusion:**

This study provides basic information for predicting the meat quality by gender using pre-deboning variables consistent with the actual grading index.

## INTRODUCTION

Carcass grade is not only a standard for production, distribution, and consumption but also for the quality index for consumer selection [1]. The main determinants of carcass grade are carcass yield and meat quality, which considerably influence price [2,3]. Under the recent domestic system, the grade of carcass yield is categorized as A, B, or C, and the yield is determined as a function of carcass weight, backfat thickness, and ribeye area. Five grades of 1++, 1+, 1, 2, or 3 are assigned for meat quality by judges’ following specific grading criteria based on marbling score, meat color, fat color, meat texture, and skeletal maturity at inspection [4].

Carcass yield is known to influence profits of distribution companies, whereas meat quality is directly related to consumer’s purchasing. In general, both carcass yield and meat quality should be simultaneously superior for a high price. However, carcass yield and meat quality grades (MQGs) are inversely proportional, suggesting that a balance between them is necessary. For this reason, various studies have investigated factors that determine either carcass yield or meat quality. With regard to meat quality, these studies have revealed that various factors, such as marbling score and water-holding capacity (WHC) [5], Warner-Bratzler shear force [6], cooking loss [7], pH [8], and refrigerating methods and aging [9], affect meat quality. For example, it has been reported that the better the carcass yield grade (CYG), the lower the MQG [10]. Even though an increase in backfat thickness has a positive effect on meat grade, there is a limitation on improving the quality grade [11]. While the backfat thickness of bulls, cows, and steers has a positive correlation with skeletal maturity and fat color in meat quality indexes, it has a negative correlation with meat color [12]. Moreover, it is reported that marbling score and backfat thickness are positively correlated, but an increase of backfat thickness by improving marbling score can cause a reduction in the carcass yield index (CYI) [13]. In addition, meat color, which is one of the determinant factors for meat quality, is a dominant consumer preference because they generally prefer scarlet-colored beef [14]. Additionally, there are studies evaluating the genetic relationship between carcass traits and carcass price [2], the effect of backfat thickness on the factor of carcass grade and price [12], contribution of yield and quality traits of Hanwoo to auction price [15], and contribution of the carcass trait to the price of Hanwoo steer [16].

Most of the previous studies have focused on the effect of meat quality and carcass yield on pricing factor in gender, and the research on the relationship between the grades of carcass yield and the meat quality according to gender is limited. Therefore, the aim of this study was to identify a distribution pattern of MQG as a function of CYI and the gender of Hanwoo (bull, cow, and steer) so that we can provide fundamental data for determining the optimum point that satisfies both yield and quality. We also attempted to identify significant deboning positions for judging meat quality through the application of discriminant analysis on pre- and post-deboning variables.

## MATERIALS AND METHODS

### Data description

The raw data from 2005 to 2008 were obtained from the National Institute of Animal Science (NIAS) in South Korea. The raw data included various pre- and post-deboning variables and recorded grades of yields, marbling, meat color, fat color, and meat quality. Because this study focused on identifying pre- and post-deboning variables discriminating carcass yield and meat quality by gender, the database was reconstructed to be suitable for a statistical significance test between the CYI and the MQG as well as a classification of meat quality [17]. The total deboning variables was 31 and included 7 pre-deboning and 24 post-deboning variables from bulls (n = 139), cows (n = 69), and steers (n = 153). In detail, the 7 pre-deboning variables included cold carcass weight, ribeye area, back-fat thickness, moisture, cooking loss, shear force, and WHC. The 24 post-deboning variables included weight of retail cuts, body fat, bone, visceral fat, tenderloin, hanging tender, strip loin, loin, chuck, top round, bottom round, blade, fore-shank, hind-shank, brisket, flank, rib, fore-leg bone, hind-leg bone, tail, knee bone, doggy bone, flank fat, and tail fat. Detailed information regarding variables and cattle is described previously [17].

### Grade of carcass yield and meat quality

The CYG is classified on a scale of A, B, or C depending on the CYI calculated by carcass weight, back-fat thickness, and ribeye area. MQG is classified on a scale of 1++, 1+, 1, 2, and 3 depending on marbling score, meat color, fat color, texture, and maturity. In this study, however, we grouped together with the 1++ and 1+ grades because the 1++ grade was too small to analyze. Basic statistics of CYI was calculated as a function of CYG.

### Statistical analysis

Discriminant function analysis (DFA) is a multivariate statistical method used for classifying multiple populations. The core of DFA is discriminant functions (DFs) determining classification points into corresponding groups [18]. DFs are created from a linear combination of the independent variables to find a new axis that maximizes the variance among groups of dependent variables (equation 1).

$$z=a+w_{1}x_{1}+w_{2}x_{2}+w_{3}x_{3}+\ldots +w_{\text{n}}x_{\text{n}}$$

Where *z* is the discriminant score; *a* is the intercept; *w*_n_ is the discriminant weights of the *n*th dependent variable; *x*_n_ is the *n*th independent variable.

Due to its classification ability, DFA has been widely applied in various fields. In particular, DFA has been applied for studies on Hanwoo, such as classifying Hanwoo genders by pre- and post-deboning variables [17], taste grading of Hanwoo beef [19], classifying geographic origins of beef [20], and analyzing images to classify tough and tender beef in bulls [21]. For this reason, this study adopted DFA to discriminate MQG using deboning parameters of Hanwoo by gender.

### Statistical software

All statistical analyses were performed using the SAS software package (ver. 9.4, SAS Institute Inc., Cary, NC, USA). Statistical significance was assumed when the p-value was less than 0.05.

## RESULTS AND DISCUSSION

### Distribution of quality grade by carcass yield index

**Figure 1:**
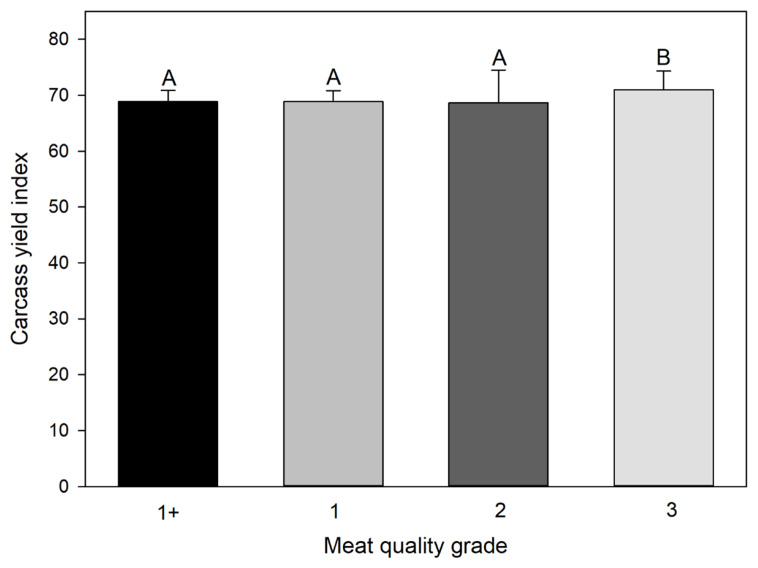
Comparison of the means of the carcass yield index grouping by the meat quality grade. Different alphabets on the bar indicate that the group mean is different, suggesting the grade 3 is significantly different from grade 1+, 1, and 2, while grade 1+, 1, and 2 are not different with each other.

**Figure 2:**
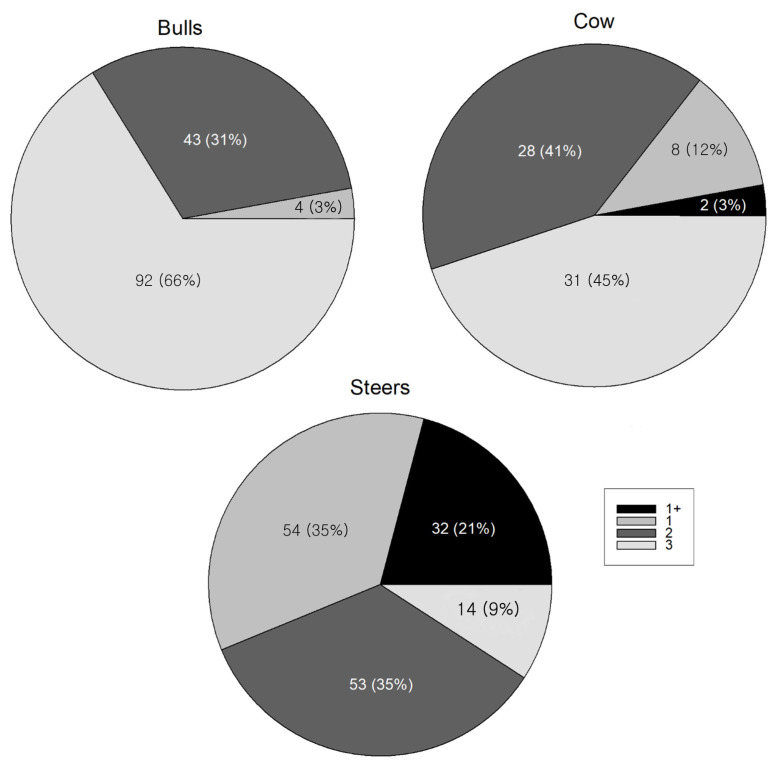
Pie chart showing the distribution of the meat quality grade according to the gender. Numbers in the pie chart indicate the number of 1+, 1, 2, and 3 grades in bulls, cows, and steers with their portions in percentage.

Analysis of variance and post-hoc test were performed using CYI to analyze its distribution by MQG. The means of CYI according to 1+, 1, 2, and 3 of MQG were 68.64±2.02, 68.85± 1.94, 68.62±5.88, and 70.99±3.32, respectively (Table 1). Grade 3 was significantly different from grade 1+, 1, and 2, whereas grade 1+, 1, and 2 were not significantly different from each other (Figure 1). MQG of 1+, 1, and 2 were correlated to CYG of B, while the MQG of 3 correlated to CYG of A. Therefore, it appeared that the lowest grade of meat quality matched the highest grade in CYI, and better quality meat was obtained at lower yields. This is consistent with a previous study that showed that a high amount of carcass limits the high level of MQG [10]. The numbers of bulls, cows, and steers in the best grade (1+ grade) of Hanwoo were 0 (0%), 2 (6%), and 32 (94%), respectively (Figure 2). In general, most of the bulls showed low-quality grade with high carcass yield, while over half of the steers were in the upper level of MQG and less than 10% of steers belonged to the 3 MQG. The majority of cows were distributed over an MQG of either 1 or 2. The above distributions are consistent with a previous study showing that a high level of meat quality is observed in the order of steer, cow, and bull [22].

### Prediction of quality grade by using pre- and post-deboning data

DFA was applied to investigate the possibility of using pre- and post-deboning data in classifying meat quality by gender. The discrimination accuracy with 7 pre-deboning variables for a total number of data regardless of gender was 64.6%, and those for 1+, 1, 2, and 3 MQG were 58.9%, 59.1%, 57.3%, and 83.2%, respectively (Table 2). The significant variables in discrimination were moisture, WHC, shear force, and backfat thickness in order. This result is consistent with the fact that the marbling score differs by the moisture contents [5,23], and the shear force is a determinant factor affecting MQG [24]. When 24 post-deboning variables were applied, the total discrimination accuracy was 59.7% and accuracy was 70.6%, 53.0%, 43.6%, and 71.5% for 1+, 1, 2, and 3 MQG, respectively (Table 3). The weights of retail cuts, bone, top round, chuck, body fat, hind-leg bone, striploin, brisket, hind-shank, rib, loin, blade, fore-leg bone, doggy bone, bottom round, fore-shank, hanging tender, and tail were significant variables affecting meat grade classification in order [25]. Overall, despite the larger number in post-deboning variables than in pre-deboning, the DF based on pre-deboning variables showed high classification ability, and this might be possible because grading carcass yield and meat quality were carried out before the deboning [26].

We further performed DFA for each gender of Hanwoo (Tables 2, 3). As a result of classification using pre- and post-deboning variables from 139 numbers of bulls, the total accuracy and its coefficient determination of DF were 80.6% and 96.5%, and 74.1% and 69.6%, respectively. Similar to using entire data, the classification ability as a function of pre-deboning data was higher than that with post-deboning data. The significant variables of pre-deboning were moisture, shear force, and cold carcass weight and classified bulls by meat quality of 1, 2, and 3 with an accuracy of 50%, 62.8%, and 90.2%, respectively. The high accuracy observed in bulls seemed that bulls had more moisture content [17,23], which significantly affected meat quality compared to others [6,12,23]. The significant variables of post-deboning data were body fat, rip, striploin, tail, hanging tender, top round, hind-shank, bone, hind-leg bone, and doggy bone. Body fat and rip only showed high correlation with DF, while the other variables showed weak correlations. The accuracies for MQG 1, 2, and 3 were 75%, 72.1%, and 75%, respectively. In the case of cows, the significant discriminant variables using pre-deboning were moisture, backfat thickness, and ribeye area, which were consistent with the indices used by domestic judges in practical grading field [26]. This consistency might lead to high accuracy in cows, resulting in 100%, 75%, 64.3%, and 74.2% for MQG of 1+, 1, 2, and 3, respectively. In contrast, post-deboning variables were not significant in meat quality classification in cows. For steers, the pre-deboning data was able to classify MQG with 56.9%, and significant variables were moisture content and shear force. Each discriminant accuracy was 68.8%, 63%, 29.6%, and 71.4% for MQG 1+, 1, 2, and 3, respectively. When it uses post-deboning data, striploin, loin, body fat, blade, and weight of retail cuts were significant variables, but accuracy was low. As expected, pre-deboning variables showed high discrimination ability; thus, the results by them could be basic information for predicting the meat quality by gender. Also, the main discriminant variables of post-deboning data were differed by gender. This might be due to the characteristic of meats, such as weight of deboning parts and the density, which were different by genders [18,27]. Thus, this analysis suggests that the application of the current post-deboning variables might not be suitable in classifying meat quality.

### Discussion on identification of optimal factors based on economics

According to the Korean Statistical Information Service [28], the steer was the most frequently appeared gender in 1+ MQG (65%) in 2018 and cow accounted for 23.4%. However, bulls were merely emerged in 1+ MQG (0.5%). The average appearance frequency in 1 MQG was in the order of cow (34.3%), steer (28.4%), and bull (3.6%), whereas he average appearance frequency in MQG 2 was bull (94.8%), cow (41.9%), and steer (11%) in order. Overall, there was a tendency for MQG to appear high in the order of steers, cows, and bulls, but MQG of bulls was remarkably low. In contrast, the average appearance frequency of A of CYG was in the order of bulls (78.1%), cow (20.4%), and steer (18.2%). For B grade in CYG, cow was most common (50.2%), while steer and bull accounted for 46.5% and 18.3%, respectively. Grade C in CYG was in the order of steer (35.2%), cow (28.9%), and bull (2.4%). Overall, these data showed that in CYG, the bull had the highest appearance frequency, whereas steer generally showed the lowest CYG. Because of the tendency of the inverse relationship between meat quality and carcass yield, Hanwoo farmers need to make a decision for a proper balance between them for maximizing profit. According to a study on the price change of carcasses by grading determinants of meat quality and yield, the effect of marbling and the carcass weight on total price was estimated by 46.3%, and 52.4%, respectively, and the marbling particularly was evaluated to contribute on auction price with 93.5% [15]. In addition, it was reported that the marbling was the significant factor affecting both carcass price and auction price in determining grid price and prediction of carcass composition [13], which indicates the importance of the meat quality factor in determining Hanwoo price. However, there was a domestic trend since 2011 that the price contribution in the meat quality has declined, whereas that of carcass yield has increased. It seemed that a decrease in price contribution of meat quality was because of the decrease in Hanwoo price which caused to increase the number of slaughtered cattle and imported beef since 2011. In addition, increased frequency of MQG 1 or higher leads to decrease in discrimination ability of marbling score [28]. Moreover, a decrease in Hanwoo supply due to national policy led to an increase in price contribution of the carcass yield [16]. Therefore, it is necessary to decide whether to concentrate on the meat quality or the carcass yield, or to get optimal profit by considering them at the same time.

To specifically discuss price factors of Hanwoo, we reviewed previous reports regarding the determinants of Hanwoo price. According to a previous study, marbling score accounted for 61.2% of the variability of the auction price [2]. In the study, for carcass price, 43.9% of the variation was mainly affected by the cold carcass weight, whereas the eye muscle area had a negligible contribution to the auction price and the carcass price. Other previous study also showed that each contribution of carcass weight, eye muscle area, backfat thickness, and marbling score was 0.17%, 2.02%, 6.52%, and 97.3% for the auction price, and 33.5%, 1.4%, 4.7%, and 60.2% for the carcass price, respectively [29]. Lee et al [3] reported that the auction price had 0.73 of the highest correlation with the marbling score, and the carcass price had 0.71 and 0.61 of correlation with the carcass weight and the marbling score, respectively. Similarly, marbling accounted for 95.4% of the auction price (R^2^ = 0.51), and 52.6% and 44.3% of the total price were attributable to marbling and carcass weight (R^2^ = 0.75), respectively [30]. Consequently, marbling and carcass weight were the most contributory factors among carcass traits and therefore, it will be possible to determine the optimal price through effective balance between these two factors. However, the livestock grading criteria has been recently revised [31]. The new grading system has developed CYI equations by genders and species and MQG grading criteria has been alleviated, which may cause increased 1++ meat. In addition, the meat color, fat color, and meat texture feed are also incorporated into the grading criteria, and 1++ meat should display its marbling score on a tag to reflect preference of consumers on grade [14]. Under the new system, it may be possible to achieve high grade in both MQG and CYI because the carcass weight reported to be inversely proportionated to MQG is managed to be a positive factor. Nevertheless, previous factors determining CYI and MQG are still used in the new system, suggesting pre/post-deboning variables can be a potential factor for discriminating grades. Moreover, this study is worthwhile to provide an example of applying multivariate analysis into livestock data.

Meat quality and carcass yield are important factors in determining the market price of Hanwoo, but they are known to be inversely proportional. For this reason, there is a demand for identifying a balance point which maximizes profits. In this point of view, this study investigated that the distribution of CYG according to MQG and analyzed variables in determining meat quality by gender. We showed that CYG was distributed in the low grade of meat quality, and the most dominant factor in meat quality was moisture, which affected the marbling score. Since the pricing factors tend to consider both carcass yield and meat quality at the same time and the tendency has systemically included in the revision of livestock grading criteria [31], it is expected to provide necessary information on judging based on distribution of carcass and its dominant factors for obtaining best profit.

## Figures and Tables

**Table 1:** Basic statistics of each carcass yield index in Hanwoo

Carcass yield index	N	Mean	Standard deviation
1+	34	68.84	2.02
1	66	68.85	1.94
2	124	68.62	5.88
3	137	70.99	3.32
Total	361	69.58	4.27

**Table 2:** Discriminant analysis of meat quality using pre-deboning variables

Items	Meat quality	Group prediction (frequency, %)								Total

		1+		1		2		3		
Total (n = 361)	1+	20	58.9	10	29.4	4	11.8	0	0	34
	1	13	19.7	39	59.1	14	21.2	0	0	66
	2	5	4.0	24	19.4	71	57.3	24	19.4	124
	3	0	10.5	2	1.5	21	15.3	114	88.2	137
Bulls (n = 139)	1+	0	0	0	0	0	0	0	0	0
	1	0	0	2	50.0	2	50.0	0	0	4
	2	0	0	9	20.9	27	62.8	7	16.3	43
	3	0	0	2	2.2	7	7.6	83	90.2	92
Cows (n = 69)	1+	2	100	0	0	0	0	0	0	2
	1	0	0	6	75.0	2	25.0	0	0	8
	2	0	0	5	17.9	18	64.3	5	17.9	28
	3	0	0	2	6.5	6	19.4	23	74.2	31
Steers (n = 153)	1+	22	68.8	8	25.0	1	3.1	1	3.1	32
	1	10	18.5	34	63.0	8	14.8	2	3.7	54
	2	4	7.5	9	17.0	21	39.6	19	35.8	53
	3	0	0	0	0	4	28.6	10	71.4	14

**Table 3:** Discriminant analysis of meat quality using post-deboning variables

Items	Meat quality	Group prediction (frequency, %)								Total

		1+		1		2		3		
Total (n = 361)	1+	25	70.6	5	14.7	5	14.7	0	0	34
	1	17	25.8	35	53.0	11	16.7	3	4.6	66
	2	17	13.7	26	21.0	54	43.6	27	21.8	124
	3	8	5.8	7	5.1	24	17.5	98	71.5	137
Bulls (n = 139)	1+	0	0	0	0	0	0	0	0	0
	1	0	0	3	75.0	1	25.0	0	0	4
	2	0	0	2	4.7	31	72.1	10	23.3	43
	3	0	0	3	3.3	20	12.7	36	75.0	92
Cows (n = 69)	1+	2	100	0	0	0	0	0	0	2
	1	0	0	7	87.5	1	12.5	0	0	8
	2	0	0	4	14.3	20	71.4	4	14.3	28
	3	0	0	2	6.5	7	22.6	22	71.0	31
Steers (n = 153)	1+	19	59.4	6	18.8	3	9.4	4	12.5	32
	1	14	25.9	29	53.7	11	20.4	0	0	54
	2	4	7.5	8	15.1	29	54.7	12	22.6	53
	3	1	7.1	1	7.1	2	14.3	10	71.4	14
